# Sensorimotor transformation via sparse coding

**DOI:** 10.1038/srep09648

**Published:** 2015-04-29

**Authors:** Ken Takiyama

**Affiliations:** 1 Tamagawa University, Machida-shi, Tokyo

## Abstract

Sensorimotor transformation is indispensable to the accurate motion of the human body in daily life. For instance, when we grasp an object, the distance from our hands to an object needs to be calculated by integrating multisensory inputs, and our motor system needs to appropriately activate the arm and hand muscles to minimize the distance. The sensorimotor transformation is implemented in our neural systems, and recent advances in measurement techniques have revealed an important property of neural systems: a small percentage of neurons exhibits extensive activity while a large percentage shows little activity, i.e., sparse coding. However, we do not yet know the functional role of sparse coding in sensorimotor transformation. In this paper, I show that sparse coding enables complete and robust learning in sensorimotor transformation. In general, if a neural network is trained to maximize the performance on training data, the network shows poor performance on test data. Nevertheless, sparse coding renders compatible the performance of the network on both training and test data. Furthermore, sparse coding can reproduce reported neural activities. Thus, I conclude that sparse coding is necessary and a biologically plausible factor in sensorimotor transformation.

When we grasp a cup on a table, we need to calculate the distance between the cup and our hands, and thus minimize the distance. In this case, inputs to our motor system include visual information (the distance between the head and the cup) and proprioceptive information (the distance between the head and the hand). To calculate the distance between the cup and our hands, the visual and proprioceptive information should be appropriately integrated, i.e., the multisensory integration is indispensable to calculating the distance^1^. To minimize the distance, our motor system needs to appropriately activate arm and hand muscles, which requires complex nonlinear computation because muscles show highly complicated responses^2^,^3^. Thus, the computation of both multisensory integration and complex muscular activity, which are the so-called sensorimotor transformations, are indispensable to proper body movements in daily life.

One solution to sensorimotor transformations is provided by a basis function framework^4^,^5^,^6^,^7^. In this framework, when a neural activity *A_i_* (*i* = 1,..., *N*, where *N* is the number of neurons) is determined by multisensory inputs *x* (e.g., visual information) and *y* (e.g., proprioceptive information), a neural network can generate an arbitrary function of *x* and *y*, not by using a form of an additive interaction, *A_i_*(*x*, *y*) = *f*(*x*) + *g*(*y*), but one of a multiplicative interaction, *A_i_*(*x*, *y*) = *f*(*x*)*g*(*y*), where *f* (·) and *g* (·) are nonlinear functions, e.g., Gaussian functions or hyperbolic tangential functions. Since the activity of neurons in the premotor cortex can be explained by the form of multiplicative interaction^8^ previous research has suggested that the motor cortex implements sensorimotor transformations in a similar manner to the basis function^6^,^7^,^8^.

On the other hand, recent advances in measurement techniques have enabled the simultaneous recording of the activities of several neurons, which has revealed an important property of neural activities: a small portion of neurons exhibits extensive activity while a large portion shows little activity, i.e., sparse coding^9^,^10^,^11^,^12^. Sparse coding has been theoretically and experimentally investigated primarily in sensory cortices and subcortical regions. Marr^13^ and Albus^14^ suggested that cerebellar learning is facilitated by sparse coding and some previous studies have suggested that the sparse coding can actually enhance adaptive control^15^ and classification learning^16^ in the cerebellum. Other theoretical studies have suggested some functional roles of sparse coding in sensory information processing: sparse coding helps reduce metabolic cost (the summation of the activities of all task-related neurons) and reproduce reported neural activities in the visual cortex^17^. As well as sensory cortices and subcortical regions, there is some experimental evidence of sparse coding in motor cortices^9^,^10^. Due to the universality of sparse coding in our neural system and some theoretically suggested functional roles of sparse coding in the cerebellar learning and sensory information processing, sparse coding is expected to play significant functional roles in sensorimotor transformations.

However, the functional roles of sparse coding in sensorimotor transformations remain unclear. In this paper, I discuss the functional roles of sparse coding in sensorimotor transformations by using a threshold linear model^18^,^19^, which can control the sparseness of neural activities using a single parameter. Computer simulations were conducted of visually guided wrist movements in various postures^8^,^20^,^21^. The inputs in this task consisted of the visual targets and posture information, whereas the outputs were the motor commands used to activate nonlinear muscle units. This means that the task required multisensory integration as well as the computation of complicated motor commands, or sensorimotor transformation. Under constant metabolic cost regardless of the sparsity of neural activities, I prove the following results: 1) sensorimotor transformation cannot be learned when a large portion of neurons exhibit extensive activity (dense coding) but can be learned in sparse coding conditions; 2) there is an optimal sparseness required to attain sensorimotor transformation; 3) The learning performance to training data as well as to test data (generalization performance) is better in sparse coding than in dense coding; and 4) neural activity in sparse coding conditions is similar to previously reported neural activities^21^ or the multiplicative interaction form.

## Results

**Figure 1:**
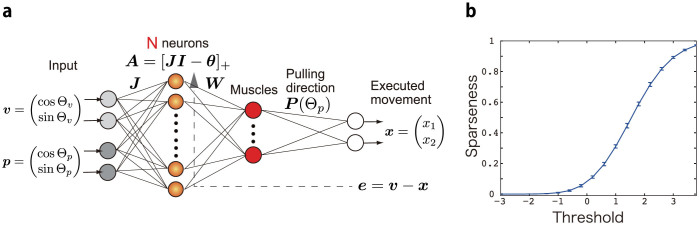
Neural network model. (a): A schematic diagram of a neural network model used to learn a sensorimotor transformation task. (b): Sparseness of neural activities. The horizontal axis denotes the threshold *θ* in equation (1) and the vertical axis denotes sparseness of neural activities. Sparseness was defined as *N*_NA_/*N*, where *N*_NA_ is the number of neurons whose activities are always 0 and *N* is the number of neurons.

Following previous studies^8^,^20^,^21^, this study focuses on visually guided wrist movements in various postures (see Figure 1a for a schematic diagram of the following computational model and the *Methods* section for the summarized procedures of the following computer simulations). In the assumed task, subjects were required to move a cursor toward a target ***v****_t_* = (cos Θ*_v_*_,*k*(*t*)_, sin Θ*_v_*_,*k*(*t*)_) on a computer screen at the *t*-th trial, where 
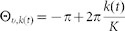
. *k*(*t*) was randomly sampled from 1,..., *K* in each trial and *K* is the number of targets (I assumed *K* = 8). The cursor movements were determined by subjects' wrist movements (e.g., the cursor moved up if a subject moved his/her wrist upward). Subjects thus needed to appropriately move their wrists to carry out the task with wrist posture ***p****_t_* = (cos Θ*_p_*_,*k*′(*t*)_, sin Θ*_p_*_,*k*′(*t*)_), where I assumed (cos Θ*_p_*_,*k*′_, sin Θ*_p_*_,*k′*_) to be 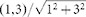 in pronation (*k*′ = 1), 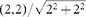 in midrange (*k*′ = 2), and 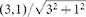 in supination (*k*′ = 3). Notably, the results were independent of these values. The target position ***v****_t_* was treated as visual information and the posture ***p_t_*** was taken to be proprioceptive information.

The task was thus to determine an executed movement ***x*** = ***P****_k_*_′(*t*)_***M*** that minimizes movement error ***e*** = ***v*** − ***x***, where ***P****_k_*_′(*t*)_ is a posture-dependent transformation matrix from muscle activity ***M*** to executed movement ***x***. Muscle activity was determined nonlinearly by neural activities ***A***: ***M*** = [***WA***]_+_, where ***W*** represents adaptable connectivities between neurons and muscles, and [·]_+_ is a linear threshold function ([*y*]_+_ = *y* when *y* > and [*y*]_+_ = 0 otherwise). The product of ***W****_t_* and ***A****_t_* was a motor command in this case because it determined muscle activity. When movement error ***e*** = ***v*** − ***x*** was observed, ***W*** was modified to minimize the squared movement error 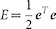 (detailed descriptions regarding the learning rule and each parameter were provided in the *Methods* section).

**Figure 2:**
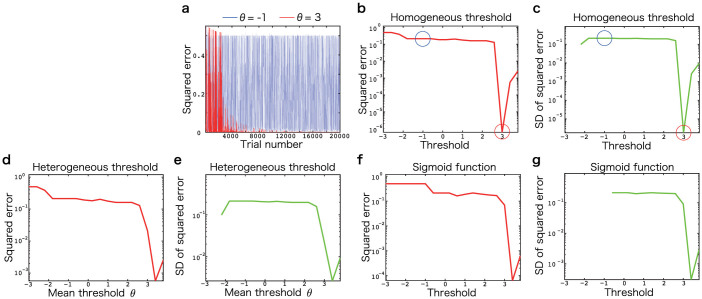
Learning ability in sensorimotor transformation task. (a): Learning curves when the threshold *θ* = −1 and 3 (these threshold values correspond to the circled values in Figures 2(b) and (c)). (b): Average squared error across 10 simulation runs. Notably, in each simulation run, the connectivity matrix *J* was fixed to be the same matrix across all values of *θ*. The horizontal axis denotes the threshold value and the vertical axis denotes log-scaled squared movement error. (c): Standard deviation of the squared error in the 10 simulation runs. The vertical axis denotes log-scaled standard deviation of the squared movement error. (d): Average squared error when threshold value is heterogeneous. (e): Standard deviation of the squared error when threshold value is heterogeneous. (f): Average squared error when neural activities were determined by a hyperbolic tangential function. (g): Standard deviation of the squared error when neural activities were determined by a hyperbolic tangential function.

To investigate the functional roles of sparse coding, a linear threshold function was assumed to represent neural activity following previous models of visual or parietal cortices^18^,^19^:
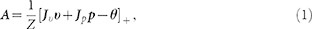
where *Z* is a normalization constant, ***J****_v_* and ***J****_p_* are fixed random matrices. The threshold ***θ*** = (*θ*_1_,...,*θ_N_*) was a crucial factor because it can control the sparseness of neural activity: when a small value of *θ_i_* was chosen, a large portion of neurons showed activity (dense coding), but only a small portion of neurons exhibited activity (sparse coding) when *θ_i_* was large. Although a homogeneous threshold value was mainly assumed, *θ_i_* = *θ*, heterogeneous threshold values did not affect any results (Figures 2d and e, see below). The above function was used because it allowed to control the sparsity of neural activities using only the parameter *θ* (Figure 1b; sparseness in this figure was defined as *N*_NA_/*N*, where *N*_NA_ is the number of neurons whose activities are always 0). The normalization constant *Z* was determined to satisfy 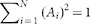, i.e., the metabolic cost was fixed across all values of *θ*. Notably, some previous studies have suggested a functional role of sparse coding is to reduce metabolic cost^17^. The normalization constant thus permitted the investigation of the functional roles of sparse coding other than reducing metabolic cost.

### Sensorimotor transformation via sparse coding

Figure 2a denotes representative learning curves in dense (blue line) and sparse coding (red line). The above-mentioned sensorimotor transformation task can be achieved only by sparse coding (Figure 2a). The task cannot be achieved by dense coding, which suggests that a linear integration of visual and proprioceptive inputs without any threshold operation is not sufficient to achieve the task. By contrast, a sparse coding network permits the learning of sensorimotor transformations possibly because of the nonlinear thresholding operation. Figure 2b shows average movement error across 10 simulation runs, and Figure 2c shows the standard deviation of the movement error. Sparse coding recorded better average movement error as well as better standard deviation than dense coding. Thus, sparse coding enables outstanding and robust learning in sensorimotor transformation. It is important to note that there was an optimal sparsity in completing the task: *θ* = 3 was the best sparsity value for this threshold linear network model and sensorimotor transformation task.

Neurons show diverse features, and it is not plausible that the threshold *θ_i_* has the same value across all neurons. Therefore, I analyzed the cases where *θ_i_* was different in each neuron (i.e., *θ_i_* = *θ* + *ξ_i_*, where *ξ_i_* was sampled from a Gaussian distribution whose mean was 0 and standard deviation was 0.1). Although optimal values of *θ* shifted from 3 to 3.4 because of the heterogeneity of the threshold value, my conclusion was not affected by this manipulation (Figures 2d and e): sparse coding enables outstanding and robust learning in sensorimotor transformation, even when each neuron has a different threshold value.

Although the significance of sparse coding in sensorimotor transformation could be found by using the threshold linear function, it remains unclear whether the significance of sparse coding is valid when the neural activities are modeled by other nonlinear functions (see the *Methods* section for a detailed description). The significance of sparse coding was found to be invariant given that neural activities were determined by a hyperbolic tangential function or a sigmoidal function (Figures 2f and g). Taken together, the significance of sparse coding in the sensorimotor transformation is a solid result. Hereafter, neural activities were determined by a threshold linear function, and the threshold values were homogeneous across all the neurons.

**Figure 3:**
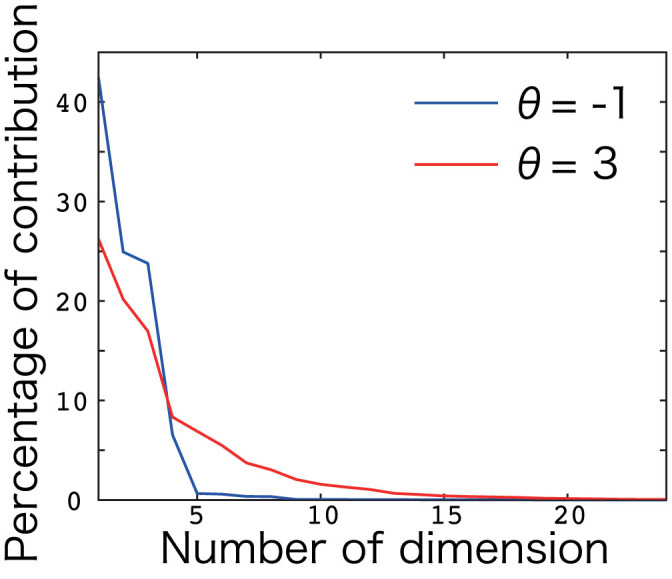
Principal component analysis of covariance matrix of neural activities. The horizontal axis denotes the number of dimensions and the vertical axis denotes the percentage of contribution, i.e., how large the *i*-th eigenvalue of the covariance matrix is compared to other eigenvalues.

Sensorimotor transformation was realized only in the sparse coding network, but the reason for this remains unclear. Since some previous studies have suggested that sparse coding can decorrelate neural activities, which can facilitate the decoding of information from the neural population^11^,^22^, I expected similar effects in sensorimotor transformation. I used a principal component analysis (PCA)^23^ for the covariance matrix of neural activities. Particularly in Equation (1), no correlation between neural activities was defined (no noise correlation was defined, and each component of ***J*** was randomly and independently sampled from a Gaussian distribution), but visual and proprioceptive information were common inputs for all neurons, thus leading to considerable correlation between neural activities. PCA enables the determination of the extent of correlation between neural activities by calculating the number of dimensional subspaces that can be observed in neural activity patterns. Since there are 24 inputs in the current sensorimotor transformation task (eight visual inputs and three proprioceptive inputs), the maximal number of dimensions is 24. If the activities of all neurons are completely independent, there is no constraint with regard to neural activities, and the number of dimension can achieve its maximal value. When the subspace consists of 24 dimensions, all 24 inputs can be encoded independently in each dimension, resulting in each input being easily distinguishable, and for it to be expected that the corresponding neural network can generate an appropriate motor command for each input. On the other hand, if the neural activities are strongly correlated, there are strong constraints of neural activities, and the number of dimensions is small. When the subspace consists of one dimension, 24 inputs are encoded confusingly in the dimension, such that each input is not easily distinguishable, and it can be expected that the neural network cannot generate an appropriate motor command for each input. As expected, the number of dimensions showing a large percentage of contributions was greater in sparse coding than in dense coding (Figure 3), thus suggesting that sparse coding decorrelated neural activities and facilitated learning. Hence, decorrelation was a reason for why a sparse coding network enabled the learning of the sensorimotor transformation task.

### Advantages of sparse coding

I also investigated the functional roles of sparse coding in the sensorimotor transformation task except for complete and robust learning. In the subsection *Sensorimotor transformation via sparse coding*, I investigated learning ability using sparse coding when *K* = 8, and sparse coding proved superior to dense coding with regard to learning ability. In general, the performance of a network on training data and that on test data cannot be compatible^23^: when trained neural networks overfit to training data, the network shows poor generalization performance on test data. I refer to this generally accepted fact about machine learning as the “incompatibility of specialization and generalization” because overfitting to training data can be regarded as a specialization of the network to the data. Based on this knowledge, one can predict that the use of sparse coding leads to worse generalization performance than that of dense coding.

**Figure 4:**
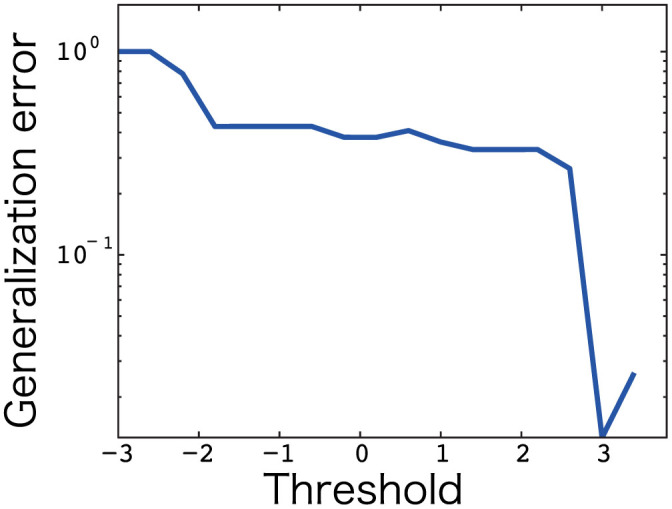
Generalization error. The horizontal axis denotes the threshold in Equation (1) and the vertical axis denotes log-scaled generalization error. The generalization error represents the averaged movement error across the 1,500 trials with 500 new visual targets and 3 postures. During the investigation of the generalization error, an adaptable matrix *W* was fixed after learning the training data.

I investigated generalization performance using dense and sparse coding with the weight matrix ***W***, which was fixed after learning, and a new target sequence ***v****_l_* = (cos Θ*_v_*_,*l*_, sin Θ*_v_*_,*l*_) and 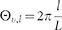 (*l* = 1,..., *L*, where *L* = 500). As shown in Figure 4, the sparse coding network showed better generalization performance than the dense coding network, which contrasted with the incompatibility of specialization and generalization. Thus, sparse coding can achieve compatibility between specialization and generalization in sensorimotor tasks.

### Multiplicatively modulated neural activities

**Figure 5:**
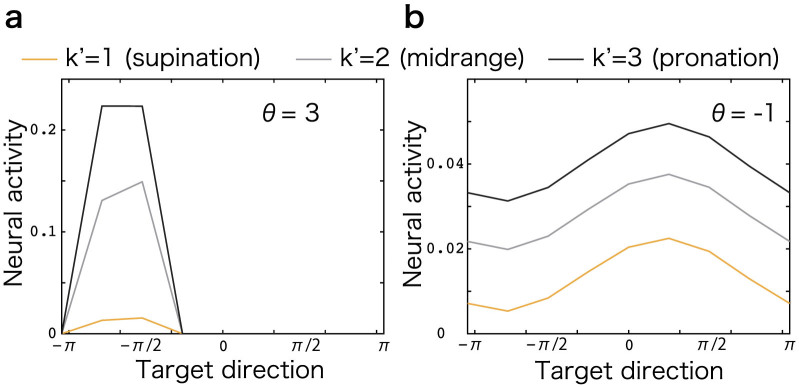
Neural activities. (a): A representative neural activity when *θ* = 3 (sparse coding network). The horizontal axis denotes visual target direction and the vertical axis denotes neural activity. Yellow, grey, and black lines denote neural activities when *k*′ = 1,2, and 3, respectively. (b): A representative neural activity when *θ* = −1 (dense coding network).

I also investigated whether sparse coding can reproduce reported neural activities. Figures 5a and 5b show representative neural activities in sparse and dense coding, respectively. In dense coding, the neural activity was additively modulated when proprioceptive information changed (i.e., *A_i_* = *f* (***v***) + *g*(***p***)), which contrasted with reported neural activities^21^. This is natural because when the threshold *θ* was low, no thresholding operation was necessary and neural activities were linearly affected by visual and proprioceptive inputs. In contrast, in the sparse coding network, neural activity seemed to be multiplicatively modulated as *A_i_* = *f* (***v***)*g*(***p***), which corresponded to previously reported neural activities. When the threshold *θ* was high, a nonlinear thresholding operation was required, which could reproduce a multiplicative interaction of visual and proprioceptive information as reported by previous neurophysiological experiments. Hence, not only did a sparse coding network attain complete and robust learning and the compatibility between specialization and generalization, it also reproduced previously reported neural activities in the sensorimotor transformation task.

### Comparisons with nonlinear network models

**Figure 6:**
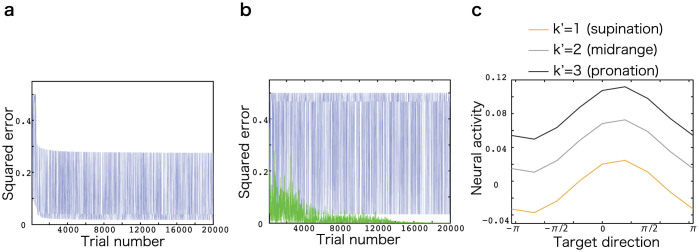
Other nonlinear networks. (a): A representative learning curve when *J* is adaptable in a hyperbolic tangential neural network. The horizontal axis denotes the number of trials and and the vertical axis denotes squared movement error. (b): Representative learning curves when both *J* and *W* are adaptable in a hyperbolic tangential neural network. The blue and green lines denote learning curves in different simulation runs. (c): A representative neural activity in a hyperbolic tangential neural network when both *J* and *W* are adaptable. The horizontal axis denotes visual target direction and the vertical axis denotes neural activity. Yellow, grey, and black lines denote neural activities when *k*′ = 1,2, and 3, respectively.

In addition to the above, I investigated whether sparse coding has advantages over other nonlinear network models. Although a linear network (dense coding) cannot facilitate learning in the sensorimotor task, some nonlinear network models are expected to do so. To show this, I first simulated a nonlinear network model where neural activities were determined by a hyperbolic tangential function, *θ_i_* = 0 (dense coding), ***W*** was fixed, and ***J*** was modified to minimize the squared movement error. These settings contrasted to the threshold linear network because ***J*** was fixed and ***W*** was modified in the network. The hyperbolic tangential network model failed to learn the sensorimotor transformation task, which suggests that nonlinearity alone in neural activities is not sufficient to learn the task (Figure 6a). Following this, I simulated a nonlinear network model in which neural activities were also determined by a hyperbolic tangential function, *θ_i_* *= * 0, and both ***J*** and ***W*** were modified to minimize the movement error. This network model could learn the sensorimotor transformation task; however, in two of the 10 simulation runs, the network model failed to learn the task (Figure 6b). These failures were possibly due to a high degree of parameter sensitivity because the initial values, including the value of each parameter, significantly affected learning performance in this network model^24^. In contrast, in sparse coding, robust learning was possible because only ***W*** was adaptable, which led to low parameter sensitivity. Furthermore, the nonlinear network models failed to reproduce multiplicatively modulated neural activities (Figures 6c). Thus, sparse coding has advantages over other nonlinear network models in that it has lower parameter sensitivity and can reproduce reported neural activities.

## Discussion

In this paper, by assuming a threshold linear network and studying visually guided wrist movements in various postures, I revealed that sparse coding is superior for learning a sensorimotor transformation task than dense coding and other nonlinear networks. When the sparsity of the neural firing was optimal, the neural network could completely and robustly learn the sensorimotor transformation task (Figure 2), by decorrelating neural activities (Figure 3). The significance of sparse coding was invariant when neural activities were modeled by a threshold linear function with the same threshold value across all neurons (Figures 2a–c), with a function with heterogeneous threshold (Figures 2d and e), or hyperbolic tangential nonlinear function (Figures 2f and g). Although overfitting to training data (specialization) and performances on test data (generalization) are incompatible in general, sparse coding successfully rendered the two compatible (Figure 4). Previous studies suggested that a functional role of sparse coding is to reduce metabolic cost^17^. However, by keeping the cost constant for both dense and sparse coding, this study revealed that sparse coding also has other significant roles in sensorimotor transformation: better learning ability, robust learning, and rendering specialization and generalization compatible in sensorimotor transformation. Furthermore, sparse coding enabled the reproduction of previously reported neural activities (Figure 5a), which were impossible by dense coding (Figure 5b) and other nonlinear neural networks (Figure 6).

The compatibility of specialization and generalization was also previously reported for a similar network model by assuming a binary neuron model and a discrimination task^25^. The study also reported that sparse coding facilitates the decorrelation of neural activities, resulting in better discrimination performance in sparse coding than in dense coding. My research here facilitated a comparison between neural activities in a sparse coding network and previously reported neural activities in sensorimotor transformation, and showed that a sparse coding network can reproduce previously reported neural activities. Hence, my work here showed another advantage of sparse coding networks, and revealed the biological plausibility of sparse coding from a different aspect: the reproduction of actual neural activities. Furthermore, although the previous and this study assumed different tasks, a discrimination and a sensorimotor transformation task, respectively, both studies found an optimal sparsity value of 0.9 (the definition of sparsity is different between the two studies). At a glance, 0.9 seems to be a magic number of sparsity; however, further analyses are necessary to rigorously discuss the optimal sparsity in various tasks. Notably, the compatibility of specialization and generalization appears to be an attractive feature of an artificial and analog neural network model, on which I focused in the current study, but further investigation is required for a competent discussion of its compatibility in more biologically plausible neural network models.

Although previous research in the area assumed that multiplicative modulation in neural activities was the crucial factor in sensorimotor transformation tasks^6^,^8^,^26^, my research here showed that sparse coding is critical to sensorimotor transformation. In fact, this network can even learn a sensorimotor transformation task after eliminating neurons whose activities are multiplicatively modulated (data not shown). Furthermore, while some studies have suggested neural implementation of multiplicative modulation^19^,^27^, I have in this paper proposed a different framework to reproduce modulation that can seamlessly connect sparse coding, the achievement of sensorimotor transformation, and reported neural activities. Furthermore, my study suggests that multiplicative modulation is a sub-phenomenon of sparse coding, and that sparse coding is an essential factor in sensorimotor transformation.

## Methods

### Learning rule

When movement error ***e****_t_* = ***v****_t_* − ***x****_t_* is observed at the 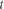-th trial, the connectivities from neurons to muscles 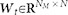 is modified to minimize the cost function 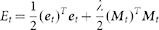, which consists of the squared movement error and the squared sum of muscle activities:
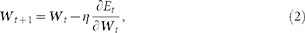
or
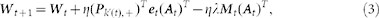
where 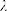 is a regularization parameter, *η* is the learning rate, and ***P****_k_*_′(*t*),+_ is a matrix whose *m*-th column was set to 0 when the *m*-th muscle activity was 0. The minimization of the squared sum of muscle activities is an important factor to discuss muscle activity^28^. On the other hand, the minimization did not play an important role in this study because I focused on the effect of the sparseness of neural activities on movement errors following the convergence of learning. 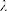 was thus set to 0 and *η* was set to 0.4. The number of trials was set to 1,000,000, but movement error had converged to a certain value by 20,000 trials. Hence, I represented movement error until 20,000 trials, as shown in Figure 2a. The average movement errors (Figures 2b–g) were calculated by averaging the movement error from the 900,000-th to the 1,000,000-th trial.

### Parameters

Each element of 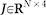 was randomly sampled from a Gaussian distribution whose mean was 0 and standard deviation was 1 for 10 times, i.e., I ran 10 simulations to calculate the average movement error shown in Figures 2b–g. The number of neurons *N* was set to 2,000. The number of neurons did not significantly affect the results because the number of neurons significantly affects the learning speed^29^. The threshold value *θ* was sampled at 18 linearly spaced points from -3 to 3.8. Actually, *θ* was sampled at 21 linearly spaced points from -3 to 5, but no neuron was active when *θ* was greater than 3.8. Thus, no learning was possible for those values of *θ*. The initial weight value ***W***_0_ was set to **0**.

The number of muscles *N_M_* represented the number of muscles and was set to 5 based on previous studies^8^,^20^,^21^. The fixed neuron-to-muscle connectivties 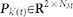 depend on posture. The (1,*i*)-th and the (2,*i*) -th components of ***P****_k_*_′(*t*)_ are defined as cos(*φ_i_*_,*k*′(*t*)_) and sin(*φ_i_*_,*k*′(*t*)_), respectively, where *φ_i_*_,*k*′(*t*)_ represents the pulling direction of the *i*-th muscle at the *k*′-th posture. The pulling direction is the direction of motion induced by muscle activation and depends upon posture. Each pulling direction was determined based on previous studies^8^,^20^,^21^.

### Summary of computer simulation

The procedures of my computer simulations can be summarized as follows. Setting the parameters ***J***, 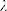, *η*, *N* to certain values, ***W***_0_ = 0, and Θ*_v_*_,*k*(*t*)_ and Θ*_p_*_,*k*′(*t*)_ to a certain value at the *t*-th trial yields the following:
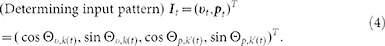

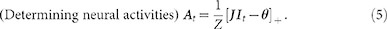

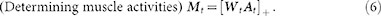

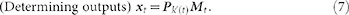

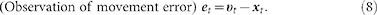

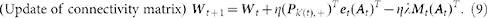


### Compared network model

In the subsection *Sensorimotor transformation via sparse coding* and *Comparisons with other nonlinear network models*, I reported the results of simulations from three hyperbolic tangential network models. In the subsection *Sensorimotor transformation via sparse coding*, neural activities were determined by
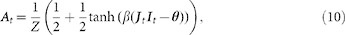
where *Z* is a normalization constant, 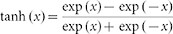, *β* determines the slope of the function, and ***θ*** determines the sparseness of the network. The normalization constant was determined in the same manner as in the threshold linear network, i.e., 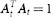. Because the maximal and minimal of tanh(*y*) is 1 and −1, neural activities are defined as non-negative values in equation (10), which is comparable to the threshold linear model. *θ_i_* = *θ* was sampled at 18 linearly spaced points from 3 to 3.8. The number of neurons *N* was set to 2000.

In the subsection *Comparisons with other nonlinear network models*, neural activities were determined by
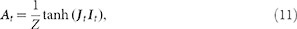
where the normalization constant *Z* was determined in the same manner as in the threshold linear network ((***A****_t_*)*^T^* ***A****_t_* = 1). Since ***W*** was modified in the threshold linear network model, only ***J*** was adaptable in one of the models, whereas both ***J*** and ***W*** were adaptable in the other. I ran 10 simulations for each model and, for the initial trial of each simulation, ***J*** was set to a zero matrix and each component of ***W*** was randomly sampled from a Gaussian distribution with mean 0 and standard deviation 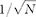 (the number of neurons *N* was set to 200 in these models).
